# Magnesium‐Encapsulated Injectable Hydrogel and 3D‐Engineered Polycaprolactone Conduit Facilitate Peripheral Nerve Regeneration

**DOI:** 10.1002/advs.202202102

**Published:** 2022-06-02

**Authors:** Zhi Yao, Weihao Yuan, Jiankun Xu, Wenxue Tong, Jie Mi, Pak‐Cheong Ho, Dick Ho Kiu Chow, Ye Li, Hao Yao, Xu Li, Shunxiang Xu, Jiaxin Guo, Qingtang Zhu, Liming Bian, Ling Qin

**Affiliations:** ^1^ Musculoskeletal Research Laboratory of Department of Orthopedics & Traumatology Innovative Orthopaedic Biomaterial & Drug Translational Research Laboratory Li Ka Shing Institute of Health Sciences The Chinese University of Hong Kong Hong Kong 999077 China; ^2^ Department of Orthopaedics & Traumatology Prince of Wales Hospital Chinese University of Hong Kong Hong Kong SAR 999077 China; ^3^ Department of Microsurgery and Orthopedic Trauma First Affiliated Hospital of Sun Yat‐sen University Guangzhou Guangdong Province 510080 China; ^4^ School of Biomedical Sciences and Engineering National Engineering Research Center for Tissue Restoration and Reconstruction Key Laboratory of Biomedical Materials and Engineering of the Ministry of Education South China University of Technology Guangzhou Guangdong Province 510006 China

**Keywords:** hydrogel, magnesium, peripheral nerve regeneration

## Abstract

Peripheral nerve injury is a challenging orthopedic condition that can be treated by autograft transplantation, a gold standard treatment in the current clinical setting. Nevertheless, limited availability of autografts and potential morbidities in donors hampers its widespread application. Bioactive scaffold‐based tissue engineering is a promising strategy to promote nerve regeneration. Additionally, magnesium (Mg) ions enhance nerve regeneration; however, an effectively controlled delivery vehicle is necessary to optimize their in vivo therapeutic effects. Herein, a bisphosphonate‐based injectable hydrogel exhibiting sustained Mg^2+^ delivery for peripheral nerve regeneration is developed. It is observed that Mg^2+^ promoted neurite outgrowth in a concentration‐dependent manner by activating the PI3K/Akt signaling pathway and Sema5b. Moreover, implantation of polycaprolactone (PCL) conduits filled with Mg^2+^‐releasing hydrogel in 10 mm nerve defects in rats significantly enhanced axon regeneration and remyelination at 12 weeks post‐operation compared to the controls (blank conduits or conduits filled with Mg^2+^‐absent hydrogel). Functional recovery analysis reveals enhanced reinnervation in the animals treated with the Mg^2+^‐releasing hydrogel compared to that in the control groups. In summary, the Mg^2+^‐releasing hydrogel combined with the 3D‐engineered PCL conduit promotes peripheral nerve regeneration and functional recovery. Thus, a new strategy to facilitate the repair of challenging peripheral nerve injuries is proposed.

## Graphical abstract

Mg‐encapsulated HA‐Pam‐Mg hydrogel demonstrates substantial shear thinning and injectability. This hydrogel combined with the 3D‐engineered PCL conduit can promote peripheral nerve regeneration and functional recovery overtime via regulating PI3K/Akt and Sema5b, demonstrating the great potential of Mg‐based biomaterials for nerve repair.

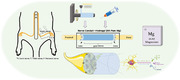

## 1 Introduction

Peripheral nerve defects are frequently caused by trauma or tumor dissection and are treated by nerve transplantation or reconstruction using various transplant materials. Autologous nerve grafting is the gold standard for treating these defects.^[^ ^1^ ^]^ However, this approach is limited by restricted autograft availability and other drawbacks, such as donor‐site morbidity, sensation loss, and neurological pain.^[^ ^2^ ^]^ Thus, a current research focus is to develop various biocompatible scaffolds using tissue engineering strategies.^[^ ^3^, ^4^ ^]^

Various biological tissues and polymers (either natural or synthetic) have been developed to bridge nerve defects and facilitate their repair.^[^ ^4^ ^]^ For instance, magnesium (Mg) and its alloys are biodegradable,^[^ ^5^ ^]^ conductive compounds that have good mechanical properties^[^ ^6^ ^]^ and superior flexibility.^[^ ^7^ ^]^ Several studies have demonstrated the potential application of Mg in peripheral nerve regeneration.^[^ ^8^ ^]^ Mg ions (Mg^2+^) are formed upon Mg degradation and are beneficial for neurite outgrowth and cell attachment.^[^ ^9^ ^]^ Moreover, oral supplementation of Mg^2+^ has been reported to enhance peripheral nerve regeneration following crush injury.^[^ ^10^, ^11^ ^]^ Mg metal filaments placed within a nerve guide conduit have been reported to improve in vivo axonal growth over a short nerve injury gap (6 mm) in a relatively short period (six weeks).^[^ ^12^ ^]^ However, they exhibited unsatisfactory outcome for a defect gap that is longer than 6 mm.^[^ ^13^ ^]^ One possible reason is that local accumulation of released Mg^2+^ ions causes neurotoxicity, suggesting that controlled Mg^2+^ release systems are required for long‐term functional recovery.^[^ ^14^ ^]^

Thus, to establish a stable system for the sustained release of Mg^2+^, our group has been studying nanocomposite hydrogels and functional nanoparticles as potential materials.^[^ ^15^ ^]^ Bisphosphonates (BPs) can coordinate with various metal ions via their inherent ability to form dynamic metal‐ligand coordination bonds and thus to form self‐assembled nanoparticles and nanostructures. Furthermore, BP‐based nanocomposite hydrogels are potential materials for various biomedical applications.^[^ ^16^ ^]^ A customized hydrogel is a carrier for cells, drugs, and other bioactive molecules. These modified nanocomposite hydrogels have unique advantages: tunable mechanical properties, a cell growth‐supporting 3D environment, and conformal filling of an irregular injury site pattern.^[^ ^17^ ^]^ Previously, we have developed Mg^2+^‐encapsulating BP‐based nanocomposite hydrogels for bone tissue engineering.^[^ ^18^, ^19^ ^]^ However, application of these hydrogels for nerve tissue engineering requires them to have similar mechanical properties and porosities as those of natural nerves. Additionally, their degradation rates are expected to match the speed of nerve regeneration.^[^ ^20^ ^]^

Typically, a guiding conduit is required to restore nerve continuity.^[^ ^21^ ^]^ Polycaprolactone (PCL) is a suitable and promising material as a novel nerve conduit^[^ ^22^ ^]^ and has been recently fabricated using 3D printing technology.^[^ ^23^ ^]^ In the present study, we developed a novel Mg^2+^‐encapsulating injectable hydrogel to promote peripheral nerve regeneration. We also systemically evaluated its nanofibrous structure, mechanical properties, and biomolecular composition. Furthermore, the mechanisms for Mg‐induced neurite outgrowth were elucidated in vitro. We also designed a 3D‐engineered PCL conduit with a tubular cavity filled with Mg^2+^‐encapsulating nanocomposite hydrogel to facilitate the repair of peripheral nerve defects in vivo. Thus, we propose an innovative and efficient technology for the repair and functional recovery of challenging peripheral nerve injuries.

## 2 Results

### 2.1 Mg‐Induced Neurite Outgrowth in Primary Dorsal Root Ganglia Neurons

**Figure 1:**
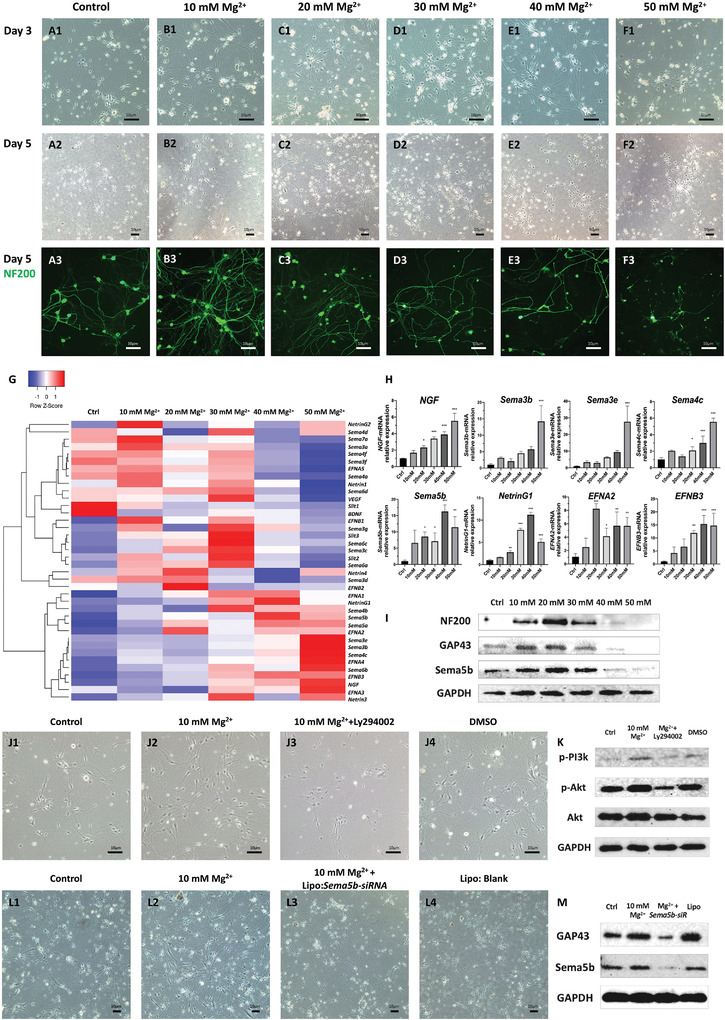
Mg^2+^ promotes neurite outgrowth in a concentration‐dependent manner by activating the PI3K/Akt signaling pathway and upregulating Sema5b expression. A–F) Primary culture of DRG neurons treated with varying Mg^2+^ concentrations (Ctrl, 10, 20, 30, 40, and 50 mM). G) Gene expression heatmap of axon guidance molecules and neurotrophic factors (*n* = 3). H) mRNA expression levels of *NGF, Sema3b, Sema3e, Sema4c, Sema5b, NetrinG1, EFNA2, EFNB3* (mean ± standard deviation; one‐way ANOVA and Tukey's *post hoc* tests, *P < 0.05; **P < 0.01 compared to the mRNA levels in the Ctrl group; *n* = 3 per group). I) Protein expression levels of NF200, GAP43, Sema5b, and GAPDH assessed by western blotting. J) Primary culture of DRG neurons treated with additional Mg^2+^ ions, LY294002, or DMSO. K) Protein expression levels of pPI3K, Akt, and p‐Akt in four groups (Ctrl, Mg^2+^, Mg^2+^ with LY294002, and DMSO). L) Primary culture of DRG neurons with additional Mg^2+^, Lipo: *Sema5b‐siRNA* or Lipo: Blank; M) Western blot results of GAP43, Sema5b expression in four groups. DRG, dorsal root ganglia; DMSO, dimethyl sulfoxide; Ctrl, control.

To determine the optimal concentration of Mg^2+^ required to promote peripheral nerve regeneration, we cultured primary rat dorsal root ganglia (DRG) neurons (**Figure** **1**A–F). Consequently, we observed that Mg^2+^ induced neurite outgrowth in a concentration‐dependent manner. Indeed, cells treated with 10 and 20 mM Mg^2+^ had higher axonal growth densities and longer axonal lengths (Figure 1B,C). However, Mg^2+^ concentration exceeding 40 mM significantly inhibited axonal outgrowth (Figure 1E,F). Remarkably, the protein expression levels of NF200 and GAP43 (Regeneration‐Associated Proteins) displayed a trend consistent with the morphological observations (Figure 1I).

### 2.2 Gene Expression Levels of Axon Guidance Molecules and Neurotrophic Factors

We generated a heatmap to evaluate gene expression levels in the six groups treated with different Mg^2+^ concentrations (Figure 1G). Our qPCR results demonstrated that the mRNA levels of *NGF, Sema3b, Sema3e, Sema4c, Sema5b, NetrinG1, EFNA2, and EFNB3* were significantly higher (Figure 1H). Moreover, Sema5b had higher protein expression, consistent with our morphological observations on axonal growth and NF200 and GAP43 protein expression levels (Figure 1I).

### 2.3 Mg‐Induced Neurite Outgrowth via the PI3K‐AKT Signaling Pathway and Sema5b

We investigated whether Mg^2+^ ions activated the PI3K/AKT signaling pathway (Figure 1J) by analyzing the PI3K, Akt, and p‐Akt expression levels in the control (Ctrl), Mg^2+^, Mg^2+^ with LY294002 (a PI3K/Akt inhibitor), and dimethyl sulfoxide (DMSO) groups by western blotting. Notably, LY294002 addition significantly decreased axonal growth density and length compared to 10 mM Mg^2+^ addition alone (i.e., without the inhibitor). It also significantly decreased the protein expression levels of pAkt/Akt and pPI3K (Figure 1K). To confirm the role of Sema5b in nerve regeneration, we applied siRNA transfection in vitro to knock down Sema5b and evaluate the growth of axons and branches (Four groups: Ctrl, 10mM Mg^2+^, Mg^2+^ with Lipo:*Sema5b‐siRNA*, Lipo:Blank). Our results showed that *Sema5b‐siRNA* significantly repressed the axon growth compared to the 10 mM Mg^2+^ without the siRNA, shown by cellular morphology (Figure 1L) and western blot (Figure 1M). These results indicated the indispensable role of Sema5b in Mg‐induced neurite outgrowth.

### 2.4 Fabrication and Characterization of Hyaluronic Acid (HA)‐Pamidronate‐Mg Hydrogel

**Figure 2:**
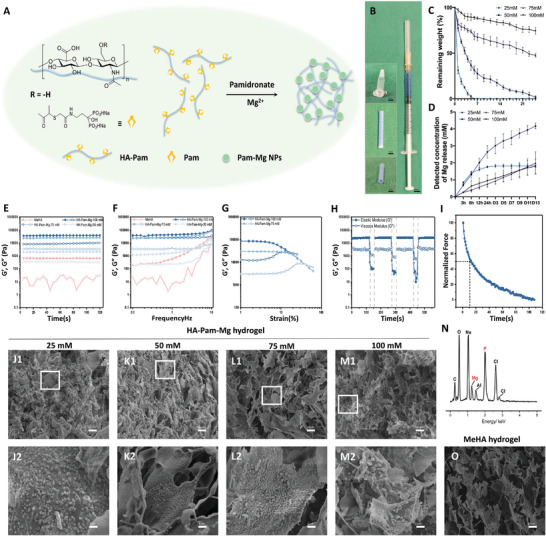
Fabrication, characterization, mechanical properties, and scanning electron microscopic observation of HA‐pamidronate‐magnesium hydrogel. A) Fabrication of the HA‐Pam‐Mg hydrogels. B) Good injectability and self‐molding properties of the HA‐Pam‐Mg hydrogels. C) In vitro degradation of the HA‐Pam‐Mg hydrogels encapsulating different concentrations of Mg^2+^ (25, 50, 75, and 100 mM). D) Accumulated Mg^2+^ release within 14 days of HA‐Pam‐Mg hydrogels. E) Time sweep at a constant shear strain of 0.1%. F) Frequency sweep from 10–0.1 Hz at a constant shear strain of 0.1%. G) Strain sweep from 0.01% to the crossover point of G′ and G″ at a constant shear frequency of 1 Hz. H) Shear‐thinning test. I: Stress relaxation test. J–M) SEM characterization of the HA‐Pam‐Mg hydrogels. N) EDS analysis of the existing elements within the hydrogel; O) SEM characterization of MeHA hydrogels for comparison. HA, hyaluronic acid; Pam, pamidronate; Mg, magnesium; SEM, scanning electron microscopy; MeHA, methacrylated HA; EDS, energy dispersive X‐ray spectroscopy.

We first grafted hyaluronic acid (HA) with pamidronate (Pam) at a substitution degree of 20% (Figures S1–S3, Supporting Information). Subsequently, we fabricated the HA‐Pam‐Mg nanocomposite hydrogel by dissolving Pam‐grafted HA (HA‐Pam, 1% w/v) and Pam (100 mM) in phosphate‐buffered saline. Following this, MgCl_2_ was added to the hydrogel solution and vortexed for 15 min (**Figure** **2**A). The hydrogel was loaded into a 1 mL syringe and easily injected through a G21 needle. Importantly, the injected hydrogel quickly adapted to the shape of the mold, which was that of a PCL conduit. It demonstrated good shear thinning, injectability, and self‐healing properties (Figure 2B). We carried out in vitro study by culturing DRG neurons with addition of HA‐Pam and/or Pam in the medium to confirm the biocompatibility of HA‐Pam and Pam, yet our findings did not reveal any promotive effect on neuron growth (Figure S4, Supporting Information). Notably, the degradation rate of the hydrogel and the release of Mg^2+^ from the hydrogel were dependent on the concentration of Mg^2+^ ions incorporated in the hydrogel (25, 50, 75, and 100 mM). Indeed, a high Mg^2+^ concentration resulted in slower hydrogel degradation and Mg^2+^ release (Figure 2C,D). Furthermore, the HA‐Pam‐Mg‐25 mM hydrogel lost approximately 68.66% of its original weight on the first day and was almost completely degraded after three days. In contrast, the HA‐Pam‐Mg‐50 mM hydrogel lost 22.54% and 87.5% of its original weight after 24 h and 15 days, respectively. Additionally, the HA‐Pam‐Mg‐75 mM and HA‐Pam‐Mg‐100 mM hydrogels degraded relatively slowly; 59.53% and 83.44% of their original weight remained on day 15, respectively. This slow degradation of HA‐Pam‐Mg‐75/100 mM hydrogels might physically block the regeneration of nerve defects. Since the Mg^2+^ release profile of the HA‐Pam‐Mg‐50 mM hydrogel matched with the speed of nerve regeneration in vivo, we, therefore, used 50 mM of Mg^2+^ concentration for subsequent experiments.

### 2.5 Mechanical Properties and Scanning Electron Microscopic Analysis of the HA‐Pam‐Mg Hydrogel

The HA‐Pam‐Mg hydrogel had good rheological properties, including a tunable storage modulus, shear‐thinning, and fast stress relaxation (Figure 2E–I). Both the time sweeps (Figure 2E) and frequency sweeps (10–0.1 Hz) at a constant shear strain of 0.1% (Figure 2F) revealed that the storage modulus of the hydrogel could be easily tuned by changing the concentration of free Pam and Mg ions. Moreover, the strain sweep displayed crossover points of G′ and G″ at a constant shear frequency of 1 Hz. This validated that the HA‐Pam‐Mg hydrogel had good shear thinning property (Figure 2G). Scanning Electron Microscopy (SEM) analysis demonstrated the porous structure of the assembled BP‐Mg nanoparticles (Figure 2J–M). Notably, we observed a peak at 1.25 keV in our EDS analysis, thereby verifying the presence of Mg^2+^ within the hydrogel (Figure 2N).

### 2.6 Fabrication, Mechanical Analysis, and SEM Analysis of PCL Nerve Conduits

**Figure 3:**
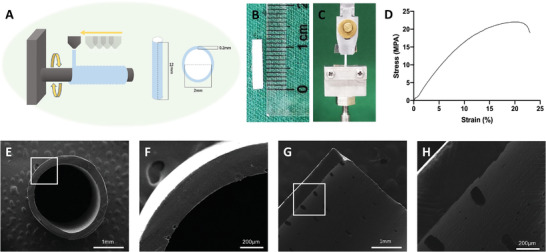
Fabrication, mechanical analysis, and scanning electron microscopy of 3D‐engineered PCL nerve conduits. A) Schematic diagram of the 3D printing process of the PCL nerve conduits. B) PCL nerve conduit with a diameter of 2 mm and a length of 12 mm. C) Mechanical analysis of PCL nerve conduits. D) Strain–stress curve. E–H) SEM analysis of the PCL conduits. PCL, polycaprolactone; SEM, Scanning Electron Microscopy.

We fabricated PCL nerve conduits using a 3D printing technique (**Figure** **3**A), wherein PCL was printed on a rotating stainless steel stick of 2 mm diameter. The conduits were then cut into 12 mm‐long pieces (Figure 3B). Subsequently, we tested the mechanical properties of the conduits and determined their ultimate force and stress to be 28.2 ± 1.79 N and 20.4 ± 1.30 MPa, respectively (Figure 3C,D). Furthermore, our SEM analysis revealed that these 3D‐engineered nerve conduits had a smooth surface and even thickness. We also observed a few pore structures in the conduits (Figure 3E–H); these formed primarily because of incomplete fusion between PCL layers. These structures were considered to be a common manufacturing limitation of fused deposition‐modeling 3D printing.^[^ ^24^ ^]^ We cultured DRG neurons on the inner surface of the conduits followed by SEM observation to further demonstrate the cytocompatibility of the 3D engineered PCL conduits and the results showed that PCL conduits were biocompatible for cell adhesion and axon growth in vitro (Figure S5, Supporting Information).

### 2.7 Functional Evaluation of Nerve Regeneration

**Figure 4:**
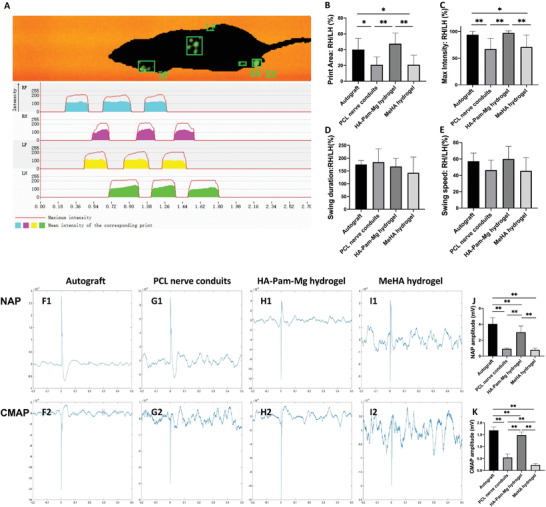
Functional recovery analysis via catwalk gait and electrophysiological assessment at 12 weeks post nerve grafts implantation. A) Footprints and gaits were automatically captured by the catwalk gait analysis system. B) Percentage print area of RH/LH. C) Percentage maximum intensity of RH/LH. D) Percentage swing duration of RH/LH. E) Percentage swing speed of RH/LH (*n* = 5). F1–I1) NAP amplitude of the autograft, PCL nerve conduit, HA‐Pam‐Mg hydrogel, and MeHA hydrogel groups. F2–I2) CAMP amplitude of the four groups. J) NAP amplitude in mV. K) CAMP amplitude in mV. Data are expressed as mean ± standard deviation. One‐way ANOVA and Tukey's post‐hoc test is performed for statistical analysis. *P < 0.05; **P < 0.01. HA, hyaluronic acid; Pam, pamidronate; Mg magnesium; PCL, polycaprolactone; NAP, nerve action potential; CAMP, compound muscle action potential.

Footprints and gaits were automatically captured in all four groups (the autograft, HA‐Pam‐Mg hydrogel, PCL nerve conduit, and methacrylated HA (MeHA) hydrogel groups; **Figure** **4**) at 12 weeks using a catwalk gait analysis system (Figure 4A). We selected the print area, maximum intensity, swing duration, and swing speed to assess motor function recovery of the rats. In this regard, a large footprint area and a high right hind foot intensity suggested good lower limb muscle strength, muscular reinnervation, and functional recovery. The autograft group had the largest footprint area and the highest right hind foot intensity. On the other hand, the HA‐Pam‐Mg hydrogel group had a larger footprint area and a greater right hind foot intensity than the PCL nerve conduit group (Figure 4B) and MeHA hydrogel group (Figure 4C). While animals in all the four groups regained a certain degree of overall motor function, differences in the swing duration or swing speed among the groups were statistically insignificant (Figure 4D,E). Furthermore, we performed electrophysiological assessment and catwalk gait analysis of rats in all the four groups to evaluate functional recovery of the regenerated nerves 12 weeks post nerve graft implantation. The autograft group had the highest nerve action potential (NAP) and compound muscle action potential (CMAP) amplitudes (Figure 4F), followed by the HA‐Pam‐Mg hydrogel group (Figure 4H), PCL nerve conduit group (Figure 4G), and MeHA hydrogel group (Figure 8I).

### 2.8 Immunofluorescence Analysis of Early Nerve Regeneration

**Figure 5:**
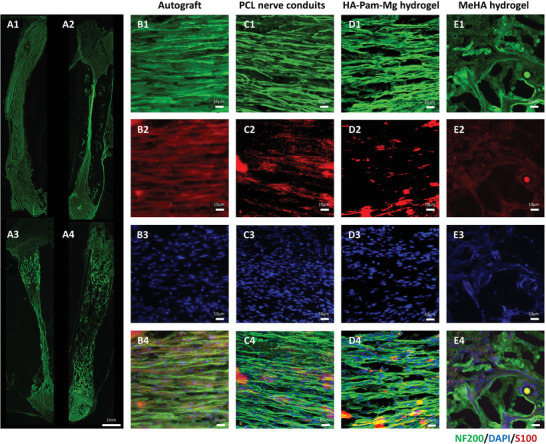
Immunofluorescence analysis of early nerve regeneration. A) Full‐length longitudinal sections of nerve samples displaying newly regenerated axons. A1–4) Autograft group, PCL nerve conduits group, HA‐Pam‐Mg hydrogel group, and MeHA hydrogel group; B–E) NF200/S100 staining of regenerated axons. Timepoint: week 2. HA, hyaluronic acid; Pam, pamidronate; Mg magnesium; PCL, polycaprolactone; MeHA, methacrylated HA.

We analyzed early nerve regeneration by performing immunofluorescence analysis of NF200 and S100 (Axon marker and Schwann cell marker) two weeks postoperatively (**Figure** **5**). Remarkably, we observed newly regenerated axons in the full‐length longitudinal sections of nerve samples obtained from all four groups (the autograft, HA‐Pam‐Mg hydrogel, PCL nerve conduit, and MeHA hydrogel groups; Figure 5A1–4). The autograft group exhibited the best matching between nerve grafts and nerve stumps (Figure 5B1–4). Interestingly, the HA‐Pam‐Mg hydrogel completely degraded within two weeks and fascicular‐like regenerated axons restored neural continuity in the Mg hydrogel group (Figure 5D1–4). Similarly, the PCL nerve conduit group also demonstrated restored neural continuity but had smaller nerve fibers (Figure 5C1–4). However, most of the hydrogel in the MeHA hydrogel group did not degrade; this could have been because of blocked nerve regeneration and reconnection. Additionally, the regenerated axons could only travel through the cracks in the hydrogel and were thus unable to form a fascicular‐like structure(Figure 5E1–4). We further performed immunofluorescence staining of Sema5b and found that Sema5b was elevated only in the Mg hydrogel group (Figure S6, Supporting Information).

### 2.9 Hematoxylin and Eosin (H&E) Staining and Immunohistochemical Analysis of Regenerated Nerves

**Figure 6:**
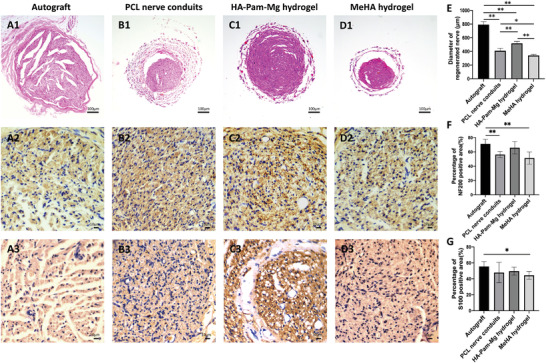
H&E staining and immunohistochemical analysis of the regenerated nerves. A1–D1) H&E staining in the autograft, PCL nerve conduit, HA‐Pam‐Mg hydrogel, and MeHA hydrogel group samples. A2–D2) Immunohistochemical analysis of NF200 staining in all four groups. A3–D3) Immunohistochemical analysis of S100 staining in all four groups. E) Diameter of the regenerated nerves (µm; *n* = 4). F) Percentage of NF200‐positive area (*n* = 4). G: percentage of S100‐positive area (*n* = 4). Data are expressed as mean ± standard deviation. Statistical analysis is evaluated by one‐way ANOVA and Tukey's post‐hoc test. *P < 0.05; **P < 0.01. H&E, hematoxylin and eosin; HA, hyaluronic acid; Pam, pamidronate; Mg magnesium; PCL, polycaprolactone.

To study regenerated nerves, we histologically evaluated the middle regions of the nerve samples (2 mm in length) harvested 12 weeks postoperatively. Furthermore, H&E staining and immunohistochemical analysis were performed to investigate the newly formed nerve fibers in each group (**Figure** **6**). We analyzed the transverse sections and measured the diameters of these nerve fibers. The autograft group had the largest diameter of statistical significance, followed by the HA‐Pam‐Mg hydrogel, PCL nerve conduits, and MeHA hydrogel groups (Figure 6E). Additionally, axonal regeneration and Schwann cell regeneration in the grafts were investigated by determining NF200‐positive axons and S100 staining, respectively. The autograft group had significantly higher NF200‐positive areas than the PCL nerve conduit and MeHA hydrogel groups. In contrast, the autograft group exhibited statistically insignificant increase in NF200‐positive areas compared to the HA‐Pam‐Mg hydrogel group (Figure 6F). Interestingly, we observed no statistical difference in the S100‐positive areas in the autograft, HA‐Pam‐Mg hydrogel, and PCL nerve conduit groups (Figure 6G).

### 2.10 Remyelination of the Regenerated Nerves

**Figure 7:**
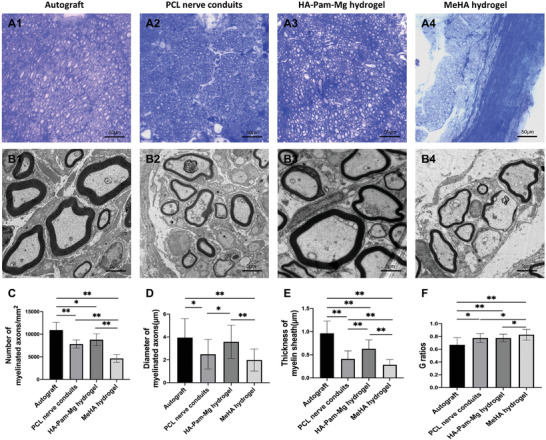
Remyelination of regenerated nerves. A) Toluidine blue staining of transverse sections of nerve grafts harvested 12 weeks post‐surgery. B) TEM analysis of the remyelinated axons. C) Number of the myelinated axons (*n* = 5). D) Diameter of the myelinated axons (µm; *n* = 5). E) Thickness of the new myelin sheath (µm; *n* = 5). F) G‐ratio (*n* = 5). Data are expressed as the mean ± standard deviation. One‐way ANOVA followed by Tukey's post‐hoc test is performed to analyze statistical significance. *P < 0.05; **P < 0.01. TEM, Transmission Electron Microscopy.

We studied remyelination of the regenerated nerves by performing toluidine blue staining (**Figure** **7**A) and transmission electron microscopy (TEM; Figure 7B). We collected nerve graft samples 12 weeks post‐surgery and subsequently observed the cross‐sections of their distal 2 mm regenerated nerves. Additionally, we counted the number of myelinated axons (Figure 7C) and measured their diameters (Figure 7D) and assessed the thickness of new myelin sheaths by TEM (Figure 7E). The G‐ratio was also calculated to determine the maturity of the myelinated axons (Figure 7F). Among the four groups, the autograft group had axons that underwent the most remyelination, as these regenerated axons were surrounded by clear, thick, and electron‐dense myelin sheaths. Axons in the HA‐Pam‐Mg hydrogel group also exhibited significantly good remyelination in terms of the number and diameter of the myelinated axons and the thickness of the new myelin sheath. Notably, certain amount of the MeHA hydrogel in the MeHA hydrogel group remained undegraded at both ends of the nerve grafts even 12 weeks post‐operation, thereby displaying the worst remyelination among all the groups. Although the PCL nerve conduit group exhibited better remyelination than the MeHA hydrogel group, the remyelination was not as efficient as that in the HA‐Pam‐Mg hydrogel group.

### 2.11 Examination of Reinnervated Triceps Surae Muscles

**Figure 8:**
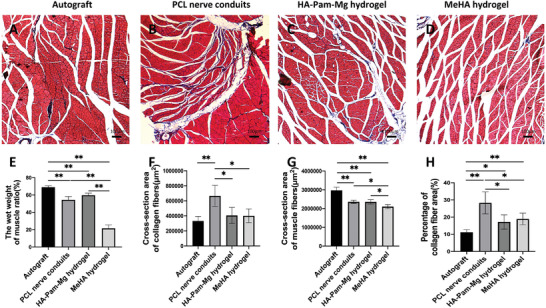
Masson's trichome staining of transverse sections of the triceps surae muscles. A–D) Triceps surae muscle samples harvested 12 weeks post‐operation from the autograft, PCL nerve conduit, HA‐Pam‐Mg hydrogel, and MeHA hydrogel groups. E) Wet weight muscle ratio (%). F) CSA of muscle fibers (µm^2^). G) CSA of collagen fibers (µm^2^). H) Percentage area of collagen fibers (%; *n* = 4). Data are expressed as mean ± standard deviation. One‐way ANOVA followed by Tukey's post‐hoc test is performed to analyze statistical significance. *P < 0.05; **P < 0.01. CSA, cross‐sectional area; HA, hyaluronic acid; Pam, pamidronate; Mg magnesium; PCL, polycaprolactone.

While muscle atrophy affected the triceps surae muscles in all four groups, this condition improved with reinnervation over time. First, we harvested triceps surae muscle samples 12 weeks post‐operation and calculated the wet weight muscle ratio (injured side/healthy side; **Figure** **8**E). We observed that while muscles in the autograft group had the least muscle atrophy compared to those in the other groups, muscles in the MeHA hydrogel group weighed the least. Furthermore, the HA‐Pam‐Mg hydrogel group showed less muscle atrophy than the PCL nerve conduit group; however, this difference was not statistically significant. Next, the morphology of the triceps surae muscles was studied by Masson's trichrome staining (Figure 8A–D). Of note, the autograft group had the largest cross‐sectional area (CSA) of muscle fibers and the smallest CSA of collagen fibers. Additionally, the PCL nerve conduit and the HA‐Pam‐Mg hydrogel groups had larger CSA of muscle fibers than the MeHA hydrogel group (Figure 8F,G). On the contrary, the HA‐Pam‐Mg hydrogel and MeHA hydrogel groups had smaller CSAs of collagen fibers than the PCL nerve conduit group (Figure 8G).

## 3 Discussion

In this study, we demonstrated the role of Mg^2+^ in promoting axonal outgrowth by activating Sema5b and the PI3K/Akt signaling pathway. We revealed that Mg^2+^ promoted neurite outgrowth in a concentration‐dependent manner. Thus, our innovative HA‐Pam‐Mg hydrogel serves as a scaffold that physically supports axonal outgrowth and induces local Mg^2+^ release. Consequently, it accelerates early nerve regeneration leading to better functional recovery from peripheral nerve injury in a rat model.

Our group has focused on the research,^[^ ^25^ ^]^ development, and application^[^ ^26^ ^]^ of Mg‐based biomaterials for musculoskeletal regeneration.^[^ ^27^ ^]^ This is because Mg is involved in several physiological processes during in vivo degradation, such as angiogenesis and osteogenesis during bone regeneration.^[^ ^28^ ^]^ Studies have also demonstrated the potential that Mg‐based alloys have in promoting bone formation.^[^ ^29^ ^]^ In the nervous system, Mg^2+^ is crucial for DNA synthesis, RNA aggregation, protein synthesis, ATPase function, plasma membrane integrity, and cellular bioenergetics.^[^ ^8^, ^30^, ^31^ ^]^ It is also essential for optimal nerve transmission and neuromuscular coordination. Indeed, several studies have demonstrated its potential application in peripheral nerve regeneration both in vitro and in vivo.^[^ ^14^, ^31^ ^]^

Several in vitro studies have reported that Mg^2+^ promotes neurite outgrowth and cell attachment. For example, Koike et al. (1983) reported that PC12 cells cultured in a fetal bovine serum‐containing medium displayed neurite formation as a response to nerve growth factor (NGF) in the presence of Mg^2+^.^[^ ^32^ ^]^ They suggested that this Mg^2+^‐dependent attachment mechanism might affect the growth cone and consequently stabilize neurite outgrowth. Turner et al. (1987) reported a similar finding: Mg^2+^ favors nerve fiber outgrowth on both collagen and laminin in NGF‐treated PC12 cells. Furthermore, they discovered that compared to Mg^2+^, Ca^2+^ had separate regulatory functions in neurite formation in PC12 cells; however, other divalent ions (Mn^2+^, Co^2+^, and Ni^2+^) did not significantly promote neurite outgrowth.^[^ ^9^ ^]^ Thus, Mg^2+^ ions possibly participate in cell proliferation. Wolf et al. (2008) demonstrated that Mg^2+^ deficiency upregulates cell‐cycle‐regulatory proteins, including p27, p21, cyclins, and CDK14.^[^ ^33^ ^]^ The membrane magnesium mitosis (MMM) model proposed by Rubin et al. (2005)^[^ ^34^ ^]^ states that release of stored Mg^2+^ is involved in protein synthesis before cell division, DNA replication, and cytoskeleton formation.^[^ ^35^ ^]^ Furthermore, observations of Vidair and Rubin (2005) supported the MMM model; they demonstrated that uracil uptake by cells is dependent on Mg^2+^. Thus, Mg^2+^ functions as an endogenous growth factor that induces uracil uptake.^[^ ^36^ ^]^

In this study, we studied the mechanisms underlying Mg‐induced neurite outgrowth in primary cultures of DRG neurons in vitro. It has been reported that the PI3K/Akt pathway plays a crucial role in various biological processes, including cancer,^[^ ^37^ ^]^ pain,^[^ ^38^ ^]^ and nerve regeneration.^[^ ^39^ ^]^ It has also been implicated in pathophysiological processes induced by Mg‐based biomaterials.^[^ ^40^ ^]^ Our results validated that the PI3K/Akt signaling pathway plays a role in Mg‐induced neurite outgrowth, as axonal growth density and length were significantly inhibited by the PI3K/Akt inhibitor LY294002. In addition to assessing transcriptional regulation in nerve regeneration, we evaluated the expression levels of axon guidance molecules and neurotrophic factors secreted nerve regeneration. Indeed, our qPCR results demonstrated that *NGF, Sema3b, Sema3e, Sema4c, Sema5b, NetrinG1, EFNA2*, and *EFNB3* had significantly higher mRNA levels. Nonetheless, only Sema5b had higher protein expression than the other molecules; this was consistent with the axonal growth trend. The role of Sema5b was further confirmed in vitro by *Sema5b‐siRNA* transfection and in vivo immunofluorescence staining, suggesting its presence and specificity, indicating the indispensable role of Sema5b as a crucial mediator in Mg‐induced neurite outgrowth. Few studies examining the function of Sema5b in the nervous system have reported that it eliminates synaptic connections in hippocampal neurons in vitro^[^ ^41^ ^]^ and acts as a repulsive cue via PlexinA1 and PlexinA3 signaling in the mouse retina and chick spinal cord.^[^ ^42^ ^]^ Another study demonstrated Sema5b to be an important mediator of spiral ganglion neuron branch refinement during development.^[^ ^43^ ^]^ However, in our study, the detailed regulative mechanism of PI3K/Akt signaling pathway for Mg^2+^ induced Sema5b expression remained unclear in vivo, which would be of interest for our future study with redesigned animal experiments and controlled variables. Another interesting observation from our study is that DRG neurons show a higher tolerance to Mg^2+^ than other types of cells in the tissue engineering studies,^[^ ^7^ ^]^ the survived cells could be analyzed by flow cytometry to provide more statistical data on the neurotoxicity effect. This phenomenon deserves a further exploration of the Mg^2+^ tolerance mechanism in different cell types to understand ion metabolism.

Notably, our observations and those of other studies on Mg‐induced neurite outgrowth highlight the potential of Mg^2+^ ions as a treatment for peripheral nerve injury. Recently, peripheral nerve injuries have increased significantly owing to the occurrence of various diseases; in fact, they have an overall incidence rate of 36.9/1 000 000 person‐years.^[^ ^44^ ^]^ Of note, several in vivo studies have demonstrated that oral supplementation of Mg^2+^ promotes peripheral nerve regeneration post‐injury. Additionally, studies have proposed that Mg^2+^ has a neuroprotective role in synaptic function; this neurological function of Mg^2+^ is primarily because of its interaction with the N‐methyl‐D‐aspartate (NMDA) receptor.^[^ ^31^ ^]^ Greensmith et al. (2000) proposed a sciatic nerve injury model^[^ ^45^ ^]^ and reported that a systemic Mg^2+^ treatment can prevent the death of motoneurons after nerve crush at birth. Furthermore, they observed that magnesium sulfate (MgSO_4_) and NMDA rescued motoneurons in axotomized rats on the 5^th^ postnatal day.^[^ ^45^, ^46^ ^]^ Gougoulias et al. (2004)^[^ ^47^ ^]^ and Kapoukranidou et al. (2005)^[^ ^48^ ^]^ also reported similar results: MgSO_4_ administration rescues motoneurons and increases the number of motor units surviving into adulthood following a sciatic nerve crush injury post‐birth. Dietary Mg^2+^ supplementation also suppresses an inflammatory response and rescues Schwann cells from apoptosis.^[^ ^49^ ^]^ For instance, Pan et al. performed sciatic nerve crush injury model in mice and fed them an Mg^2+^ diet 3 weeks before and 4 weeks after the injury. Indeed, a high‐Mg^2+^ diet resulted in better functional recovery. This anti‐inflammatory effect was consistent with a significantly improved neurological function. They also reported that a high‐Mg^2+^ diet significantly increased the expression of the magnesium transporter genes CNNM2, MagT1, and SCL41A1 in the crush injury model.^[^ ^11^ ^]^

According to the Sunderland classification, nerve injuries of the third degree or above require surgical intervention, including bridging nerve defects with a nerve repair material or performing nerve transplantation to restore continuity and reconstruct normal function.^[^ ^50^ ^]^ An ideal nerve graft for nerve defect treatment should satisfy several biological^[^ ^51^ ^]^ and physicochemical requirements, including biocompatibility, biodegradability, modified biomechanical properties,^[^ ^52^ ^]^ matching microstructure,^[^ ^53^ ^]^ and providing a 3D cell growth microenvironment.^[^ ^54^ ^]^ Degradable Mg and its alloys are potential biomaterials for bridging nerve defects owing to their advantageous flexibility, ideal degradation time, and physical strength.^[^ ^14^ ^]^ Mg metal filaments placed within a nerve guide conduit have been reported to improve axonal growth over a short nerve injury gap (6 mm) in a relatively short duration (six weeks).^[^ ^13^ ^]^ Similarly, Li et al. (2016) demonstrated the use of biodegradable Mg metal wire for promoting the regeneration of compressed sciatic nerves in rats.^[^ ^55^ ^]^ They observed that Mg filament implants potentially improved the repair of injured peripheral nerves. However, unsatisfactory results were obtained for a large gap (15 mm) at 16 weeks post‐repair. One possible reason can be the local accumulation of Mg^2+^ released directly from the Mg alloy, resulting in neurotoxicity. These observations were validated in vitro in the present study.

Hence, we used a nanocomposite hydrogel as a scaffold for peripheral nerve regeneration. Since these hydrogels are available as various nanostructures, such as nanoparticles, nanorods, and nanofibers, they have several advantageous properties:^[^ ^56^ ^]^ biocompatibility, tunable mechanical properties, porosity, controllable biodegradation, and drug delivery.^[^ ^57^ ^]^ For instance, Wu et al. (2017) developed a nanofiber hydrogel containing the self‐assembling peptide RADA16‐Mix for peripheral nerve regeneration.^[^ ^58^ ^]^ Lin et al. (2020) used a hydrogel derived from a porcine decellularized nerve to repair 15 mm sciatic nerve defects.^[^ ^59^ ^]^ Recently, Rao et al. (2020) developed an aligned chitosan fiber hydrogel grafted with bioactive peptide as a nerve conduit filler to repair sciatic nerve injury.^[^ ^60^ ^]^

BP‐based nanocomposite hydrogels can form intrinsic metal‐ligand coordination bonds and thus can coordinate with various metal ions to form self‐assembled nanoparticles and nanostructures.^[^ ^61^ ^]^ These hydrogels have been applied in various biomedical fields, including drug delivery and tissue regeneration. Zhang et al. (2017) developed an HA‐BP‐Mg nanocomposite hydrogel that stably release Mg^2+^ in a controlled manner at bone defect sites to promote osteogenesis.^[^ ^62^ ^]^ Additionally, BP‐based injectable hydrogels combined with the drug release of dexamethasone have significantly enhanced bone regeneration.^[^ ^18^ ^]^ Owing to their desirable properties, such as self‐healing, injectability, and tunable mechanical properties, modified BP‐based hydrogels have great potential in nerve tissue engineering. However, achieving a significant effect in the critical nerve defect model was challenging by optimizing the appropriate density, porousness, degradation rate, and other required properties. In addition, the outcome for peripheral nerve regeneration is also closely related to microsurgical techniques, mechanical properties of the hydrogel, and optimal Mg^2+^ release from the hydrogel.

This study is the first of its kind to report the use of Mg^2+^‐encapsulating hydrogels as tissue engineering scaffolds and delivery systems that promote peripheral nerve regeneration. Thus, our study provides a new direction for the application of Mg and Mg‐based biomaterials in the nervous system. The mechanical properties of the HA‐Pam‐Mg hydrogel varied with the concentration of Mg^2+^ ions. We selected the hydrogel encapsulating 50 mM of Mg^2+^ for further in vivo studies, as it stably released Mg^2+^ and exhibited a desirable degradation duration. We prepared four groups of nerve grafts to repair sciatic nerve defects in rats, with an autograft designated as a positive control. Subsequently, we compared PCL nerve conduits filled with or without different hydrogels to demonstrate the role of hydrogels in nerve regeneration. On the other hand, we compared the HA‐Pam‐Mg hydrogel group with the MeHA hydrogel group to understand how a specific formula (especially with or without magnesium) affected the overall results. However, there was certainly a knowledge gap between in vitro and in vivo studies in our study and other studies using similar local drug delivery strategies where we were not able to detect the actual real‐time concentration of Mg^2+^ released from the hydrogel after implanted in vivo. The HA‐Pam‐Mg hydrogels exhibited good injectability and self‐molding properties and filled the nerve conduit cavities when injected in vivo. After the injury, the growth speed of the nerve is approximately 1mm/day, and nerve regeneration could be detected as early as the third‐day post‐injury in rats.^[^ ^63^ ^]^ In our study, the nerve defect was 10 mm, thus the initial 2 weeks were the critical time window to evaluate early nerve regeneration and the bridging effect of our nerve grafts. Owing to their modified formula, the HA‐Pam‐Mg hydrogels degraded upon Mg^2+^ release and got completely degraded within 2 weeks post‐injection, thus it is reasonable to conclude that HA‐Pam‐Mg hydrogels meet the requirements of the nerve growth in vivo. Compared to the PCL nerve conduit and MeHA hydrogel groups, the HA‐Pam‐Mg hydrogels released Mg^2+^ ions that promoted early nerve regeneration without physically hindering axonal outgrowth. Among the four groups, the autograft group exhibited the best results in terms of nerve regeneration and functional recovery, whereas the HA‐Pam‐Mg group demonstrated better results than the PCL nerve conduit and MeHA hydrogel groups. Surprisingly, the MeHA hydrogel group had the worst performance; this could be because of the slow degradation of the MeHA hydrogel, which physically blocked the defect gap for nerve regeneration. The intervention efficacy of HA‐Pam‐Mg was a result of the combined effect of Mg^2+^ and other biological macromolecules,^[^ ^64^ ^]^ apart from the Mg^2+^‐induced neurite outgrowth effect that we confirmed from our in vitro study, the porous structure formed by hydrogel could also provide structural support for axon growth which is one of the recognized advantages of hydrogel application in tissue engineering.^[^ ^65^ ^]^ However, since HA‐Pam‐Mg hydrogel fully degraded within 2 weeks, we could not delineate the specific effect of Mg^2+^ contributing to nerve regeneration, remyelination, and functional recovery on week 12.

Overall, our study is novel in emphasizing the role of Mg in peripheral nerve regeneration. Our work provides a new dimension in using Mg and Mg‐based biomaterials for their applications in the nervous system. Taking full advantage of the ability of Mg^2+^ on promoting nerve regeneration, PCL conduit’ guiding role, and hydrogel's scaffold role, we promoted nerve regeneration and functional recovery through these comprehensive biological actions. Also, from both in vitro and in vivo results, the mechanism of magnesium promoting nerve regeneration was investigated in detail, and the involvement of axon guidance cues Sema5b in nerve regeneration was reported for the first time. PCL conduits exerted steady effect as alternative nerve grafting materials for peripheral nerve injury. While the 3D‐printing technique is an easy and rapid way to fabricate customized nerve conduits, the 3D structure presented here (single tunnel conduit) is still relatively simple. This is further limited by the accuracy of the equipment used in this study. For future studies, an ideal nerve conduit must be designed such that it achieves nerve morphology matching and mimics the microstructure at the nerve fascicle or endoneurium level. Sine the HA‐Pam‐Mg hydrogel can be easily fabricated at a low cost and can be injected at the injury site in practice, future studies can explore its ability to possibly augment cells, drugs, or other bioactive molecules; thus, this hydrogel has a great potential for clinical translation.

## 4 Conclusion

In this study, we developed a novel Mg‐encapsulating injectable hydrogel for peripheral nerve regeneration and disclosed the underlying mechanism of Mg‐induced neurite outgrowth. In this regard, we demonstrated that Mg^2+^ promoted axon outgrowth in a concentration‐dependent manner by activating the PI3K/Akt signaling pathway. Additionally, Sema5b was also involved in nerve regeneration. The HA‐Pam‐Mg hydrogel demonstrated substantial shear thinning and injectability. This hydrogel and the 3D‐engineered PCL conduits promoted peripheral nerve regeneration and functional recovery over time. Thus, we demonstrate the excellent potential of Mg‐based biomaterials for application in the nervous system.

## 5 Experimental Section

### Study Design

The optimal concentration of Mg^2+^ (Ctrl, 10, 20, 30, 40, and 50 mM) required for nerve regeneration in primary cultures of rat DRG neurons was first determined. Additionally, the expression levels of axon guidance molecules (four families: netrins, slits, ephrins, and semaphorins) were quantified and neurotrophic factors (NGF, brain‐derived neurotrophic factor (BDNF), and vascular endothelial growth factor (VEGF)) in DRG neurons on day 7 were quantified via qPCR and western blotting. Furthermore, gene expression in DRG neurons cell culture (Four groups: Ctrl, 10 mM Mg^2+^, Mg^2+^ with PI3K/Akt inhibitor LY294002, DMSO) (Four groups: Ctrl, 10mM Mg^2+^, Mg^2+^ with Lipo: *Sema5b‐siRNA*, Lipo: Blank) were analyzed via western blot. The Mg‐encapsulating hydrogel was composed of HA grafted with Pam. To fabricate the HA‐Pam‐Mg injectable nanocomposite hydrogel, HA‐Pam (1% w/v) and Pam (100 mM) were dissolved in PBS, and to this hydrogel solution MgCl_2_ was added and vortexed. Subsequently, PCL nerve conduits were prepared by a 3D printing technique. The authors then established a 10 mm sciatic nerve defect model in vivo in 56 8‐week‐old rats. These rats were divided into four groups and their potential to promote nerve defect repair was compared. These groups were the autograft, PCL nerve conduit, PCL nerve conduits with the HA‐Pam‐Mg hydrogel, and PCL nerve conduits with the MeHA hydrogel (commonly used without Mg as a negative control) groups. The rats were euthanized at 2 and 12 weeks post‐surgery for sample collection. Thereafter, histological staining, immunohistochemical staining, TEM, walk gait analysis, and neuro‐electrophysiological monitoring were performed with a focus on the evaluation of nerve regeneration and functional recovery.

### Primary DRG Neuron Culture

DRG were collected from 2–4‐week‐old Sprague‐Dawley (SD) rats. Bilateral DRG were cultured in vitro in a 35 mm culture dish and studied under a microscope. Next, neuronal fibers connected to the DRG were removed and sensory neurons were isolated from the DRG by sequential digestion using papain and type I collagenase (1 mg mL^−1^) for 3 h, as reported previously.^[^ ^66^ ^]^ Thereafter, 5000 cells mL^−1^ of these isolated cells were cultured in a Neurobasal medium (A358290, Thermo Fisher Scientific) containing 2% B27 (A3582801, Thermo Fisher Scientific), 0.3% L‐glutamine (25030081, Thermo Fisher Scientific), 100 ng mL^−1^ NGF (N2513, Sigma‐Aldrich), 1% PSN, 10^−5^ M fluorodeoxyuridine (F0503, Sigma‐Aldrich), and 10^−5^ M uridine (U3750, Sigma‐Aldrich). These cells were cultured in a six‐well plate coated with poly D‐lysine hydrobromide (50 ng mL^−1^; Life Technologies). The following day, the medium was replaced with one containing different treatment reagents; subsequently, the medium was refreshed every two days. SiRNA transfection (siRNA ID#: s155625, Thermo Fisher Scientific) (Sequence (5′→3′): Sense: CCGUGGGUGUCUAACUUCAtt; Antisense: UGAAGUUAGACACCCACGGct.) was applied in vitro to knockdown the expression of Sema5b.

### Quantitative Real‐Time PCR (qPCR)

Total RNA was extracted from the primary DRG neurons on day 7 using the TRIzol reagent (15596026; Invitrogen, Waltham, MA, USA). The concentration and purity of the extracted total RNA were then determined using a NanoDrop 2000 (Thermo Fisher Scientific, USA) and reverse‐transcribed into cDNA using the First Strand cDNA kit (Takara, Dalian, China). Real‐time quantitative PCR was performed with cDNA as a template using TF pack power SYBR Green qPCR SuperMix‐UDG.^[^ ^67^ ^]^ The gene expression levels of axon guidance molecules (four families: netrins, slits, ephrins, and semaphorins) and neurotrophic factors (NGF, BDNF, and VEGF) were assessed.^[^ ^68^ ^]^ The sequences of primer used in this experiment are listed in Table S1, Supporting Information. Last, a gene expression heatmap was generated (www.heatmapper.ca/expression/), wherein expression levels of the target genes were normalized to that of *GAPDH*

### Western Blotting

DRG neurons cultured for 7 days were lysed or dissolved in the radioimmunoprecipitation assay buffer containing a proteinase/phosphatase inhibitor mixture. Thereafter, proteins of intact DRG neurons were isolated for western blot analysis, as per a previously published protocol.^[^ ^69^ ^]^ These proteins were then separated on an SDS‐polyacrylamide gel, and subsequent steps were performed in accordance with a previous protocol.^[^ ^70^ ^]^ Antibodies against the following proteins were employed: GAP43 (1:500, A16857, Abclonal), NF200 (1:500, N0142, Sigma‐Aldrich), Sema5b (1:500, A13819, Abclonal), pPI3K (1:500, 4292S, Cell Signaling Technology), pAkt (1:500, 4060S, Cell Signaling Technology), and Akt (1:500, 4691S, Cell Signaling Technology). The expression levels of the target proteins were analyzed normalized to that of GAPDH.

### Preparation of the HA‐Pam‐Mg hydrogels

The HA‐Pam‐Mg nanocomposite hydrogel was synthesized as per a previously published protocol with some modifications.^[^ ^18^ ^]^ The Pam‐grafted HA (1% w/v) and Pam (100 mM) were dissolved in PBS. To this solution, MgCl_2_ (500 mM) was added and vortexed. Thereafter, the mixture was left undisturbed for 15 min to ensure its complete gelation. The Pam‐Mg nanoparticles functioned as cross‐linkers that stabilized the HA‐Pam polymeric network, thereby forming the HA‐Pam‐Mg nanocomposite hydrogel. To specify the effect of the individual component within the hydrogel and determine whether they presented positive effect on nerve regeneration, in vitro culture of DRG neurons were carried out with additional Mg^2+^, HA‐Pam, and/or Pam in the medium (5 groups: Ctrl; 0.05% HA‐Pam (w/v); 5 mM Pam; 0.05% HA‐Pam (w/v) + 5 mM Pam; 5 mM Mg^2+^.), where axon growth was observed on day 5.

### Degradation and Mg Release of the HA‐Pam‐Mg Hydrogels

To test the degradation profiles of the HA‐Pam‐Mg hydrogel, different concentrations of Mg^2+^ were encapsulated (25, 50, 75, and 100 mM) within the hydrogel. Next, the initial weights of the hydrogels were measured and they were subsequently transferred and incubated in 1 mL PBS at 37 °C. Once the upper solution was absorbed, the remaining weight of the hydrogel was measured every day till day 7 and every other day from days 9–25. The Mg^2+^ release profiles of the HA‐Pam‐Mg hydrogels were evaluated according to a published protocol.^[^ ^71^ ^]^ In this regard, the HA‐Pam‐Mg hydrogel encapsulating different concentrations of Mg^2+^ (25, 50, 75, and 100 mM) was prepared and they were incubated in 500 µL PBS at 37 °C. The upper solution (500 µL) was collected at different time points (3, 6, 12, and 24 h and days 3, 5, 7, 9, 11, and 13). The concentration of Mg^2+^ was measured by inductively coupled plasma‐optical emission spectrometry (ICPE‐9820, Shimadzu, Japan); a known concentration of MgCl_2_ was used as a standard.

### Rheological Properties of the Hydrogels

All rheological tests were conducted using a rheometer (Kinexus, Lab+, Malvern, U.K.) comprising a 20 mm plate rotor. The time and frequency (10–0.1 Hz) sweeps were performed at 0.1% constant shear strain, and the strain sweeps were scanned from 0.1–100% at 1 Hz constant frequency. Shear‐thinning tests were performed with three loops of a low shear strain (0.1%) and a high shear strain (100%). Stress relaxation tests were performed by applying a 20% compression strain in a short period (≈0.5 s), followed by maintenance of this strain for 30 min to record stress attenuation.

### SEM Analysis of the Hydrogels

First, the HA‐Pam‐Mg and MeHA hydrogels were fixed in 2.5% glutaraldehyde for 6 h. Following this, they were rinsed thrice (30 min per rinse) in deionized water and then dehydrated in a graded series of alcohol (30%, 50%, 75%, and 100% ethanol) for 30 min per rinse. The hydrogel, balanced with 100% ethanol, was subsequently transferred and dried using a critical point dryer. This dried hydrogel was cut and trimmed to expose its cross‐sectional structures. It was then coated with a thin layer of a platinum and gold mixture to enhance its conductivity and contrast. The micromorphology of the hydrogels was observed under a scanning electron microscope (HITACHI S‐8010) at an accelerating voltage of 5 keV. The structural parameters of the samples were assessed using an image analysis software (ImageJ; National Institutes of Health). Eventually, an EDS analysis was performed to confirm the presence of Mg^2+^ within the hydrogel.

### Preparation and Characterization of 3D‐Engineered PCL Conduits

PCL conduits were fabricated using a 3D printing technique (Shanghai Graphic Design Information Co., Ltd., China). Briefly, a PCL filament of 200 µm diameter was printed on a rotating (60 rad min^−1^) stainless‐steel disk of 2 mm diameter for 30 min. Thereafter, the conduits were removed from the disk and vacuum‐dried for 24 h. Following this, they were cut into 12‐mm long pieces and imaged using a scanning electron microscope (Hitachi S‐3400N). The mechanical properties of the PCL nerve conduits were evaluated by tensile testing (Instron 3345, USA, 100N load cell) at a strain rate of 10 mm min^−1^. To further demonstrate the cytocompatibility of the 3D engineered PCL conduits, DRG neurons were cultured on the inner surface of the conduits followed by SEM observation on day 3 after implanted in vitro.

### Animals and Surgical Procedures

**Figure 9:**
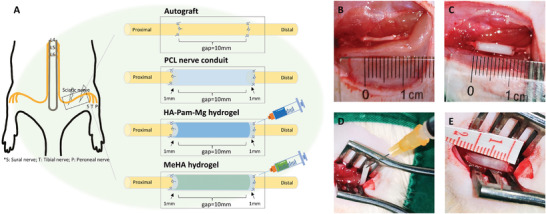
Schematic illustration of animal surgery. A) Surgical procedures performed on SD rats belonging to four groups, that is, the autograft, PCL nerve conduit, HA‐Pam‐Mg hydrogel, and MeHA hydrogel groups. B) Repair of a nerve defect using autografts. C) Repair of a nerve defect using PCL nerve conduits. D,E) Surgical procedures in the HA‐Pam‐Mg hydrogel and MeHA hydrogel groups, wherein first one nerve stump is sutured, followed by injection of the hydrogels into the nerve conduit cavities. HA, hyaluronic acid; Pam, pamidronate; Mg, magnesium; SEM, scanning electron microscopy; MeHA, methacrylated HA.

56 female SD rats (age: 2 months; weight: 200–250 g) were obtained from the Laboratory Animal Services Center of the Chinese University of Hong Kong (Hong Kong, China). All animal experiment protocols performed in this study followed the principles for the care and use of laboratory animals approved by the Animal Experimentation Ethics Committee of the Chinese University of Hong Kong (Ref. No. 19/251/MIS5‐C). These rats were randomly divided into four groups: autograft, PCL nerve conduit, HA‐Pam‐Mg hydrogel, and MeHA hydrogel (**Figure** **9**A).

The rats were kept under general anesthesia (ketamine 75 mg kg^−1^ body weight + xylazine 10 mg kg^−1^ body weight) and underwent microsurgery performed by a single well‐trained surgeon. First, a rat was surgically draped, and the surgical site was sterilized (lateral side of the right thigh, an incision of approximately 4 cm, parallel to the lower edge of the femur), including the right back of the hip and thigh. An approximate 4 cm incision was made on the lateral side of the right thigh and the sciatic nerve was carefully exposed. These surgical procedures were performed under a surgical microscope. In the autograft group, a 10 mm long section of the sciatic nerve was excised, inverted, and re‐implanted. Moreover, the autografts were sutured to the epineurium of the proximal and distal nerve stumps using a 10‐0 monofilament nylon suture. For the PCL nerve conduit, HA‐Pam‐Mg hydrogel, and MeHA hydrogel groups, the proximal end of the exposed sciatic nerve was cut first, following which the nerve stumps were moved 1 mm to the proximal end of the 12‐mm conduit (leaving a 10 mm gap). They were then fixed in place by a single 10‐0 nylon suture. The HA‐Pam‐Mg or MeHA hydrogel was injected into the nerve conduit after one end of the nerve conduit was sutured to the nerve stump. This procedure was repeated on the distal end of the nerve. The diameter of the conduit (approximately 2 mm) matched that of the sciatic nerve. Last, the skin wound was closed with 4‐0 silk sutures (Figure 9B–E).

### Functional Evaluation of Nerve Regeneration

The functional recovery of regenerated nerves was evaluated using a catwalk gait analysis system^[^ ^72^ ^]^ (Noldus Information Technology, The Netherlands). Briefly, the rats were placed on the right side of a runway consisting of a glass surface and black plastic walls. They were trained to traverse to the left end of the runway, where food pellets were kept as reward. Furthermore, they were filmed from underneath while crossing the runway in a darkened room, which enabled a good contrast between paw prints and the rest of the body. The footprints and gaits of the rats were automatically captured by the catwalk gait analysis system. Print area, maximum intensity, swing duration, and swing speed were calculated to determine the motor functional recovery of the rats.

In addition, electrophysiological analysis was used to evaluate the functional recovery of regenerated nerves at 12 weeks post‐operation. Five rats from each group were anesthetized 12 weeks after surgery. After exploring the repair site of the sciatic nerve electrical stimuli were applied to the nerve trunk at the proximal end of the graft site and the resultant signals were detected by the TDT electrophysiology system (Tucker‐Davis Technologies, Alachua, FL, USA). Of note, the stimuli were provided in pulses (stimulus intensity = 0.7 mA; duration = 5 ms; filter: 1–10 HZ, 10–1000 HZ). First, a signal‐receiving electrode was placed at the distal end of the graft site to detect the NAP. Next, CMAPs were recorded by placing the signal‐receiving electrode at the triceps surae muscles.^[^ ^73^ ^]^ To reduce external interference in the entire testing process, it was ensured that the testing environment was noiseless and carefully maintained the temperature and humidity of the nerves and electrodes. Raw data were imported into MATLAB (Mathworks, Natick, MA, USA) for quantitative analysis.

### Immunofluorescence Analysis of Early Regenerated Axons

2 weeks post‐surgery, four rats from each group were sacrificed by intraperitoneal pentobarbital injection (25%, lethal dose). Subsequently, the nerve grafts were harvested and cut longitudinally into 7 µm‐thick sections using a freezing microtome (CM3050S; Leica Microsystems). Axons were then visualized using an anti‐neurofilament NF200 antibody (1:200; N0142; Sigma‐Aldrich) and a secondary Alexa Fluor 488‐conjugated goat anti‐rat IgG antibody (1:1000; A11006; Invitrogen). Additionally, Schwann cells were visualized using an S100b antibody (1:200; HPA015768; Sigma‐Aldrich) and a secondary antibody Alexa Fluor 594‐conjugated donkey anti‐rabbit IgG (1:1000; R37119; Invitrogen). Sema5b expression was visualized using the S100b antibody (1:200; PA5‐66850; Thermo Fisher Scientific), and the secondary antibody Alexa Fluor 488‐conjugated donkey anti‐rabbit IgG (1:1000; R37118; Invitrogen). These dually stained sections were observed under a fluorescence microscope (Leica Q500MC; Leica, Germany).

### Histological Evaluation and TEM Analysis of Regenerated Nerves

At 12 weeks post‐operation, regenerated nerves were dissected and harvested after perfusing 4% paraformaldehyde (a fixing reagent) through the circulatory system of the rats. Moreover, nerve specimens were obtained from the contralateral uninjured sciatic nerve and a nerve graft from the same animal. This nerve graft was divided into three equal parts: the proximal part towards the spine, the middle part, and the distal part towards the knee.^[^ ^73^ ^]^ The middle region was cut into 7 µm‐thick cross‐sections (CM3050S, Leica, Wetzlar, Germany). Next, the sections were stained with H&E and immunohistochemical analysis of NF200 (1:200; N0142; Sigma‐Aldrich) and S100 (1:200; HPA015768; Sigma‐Aldrich) expression was performed. Images from five random fields for each section were analyzed, and four samples from each group were analyzed statistically. The percentage of NF200‐positive axon staining and that of S100‐positive area ratio were quantified using the ImageJ software.

Furthermore, distal regions of the nerve grafts were dissected for toluidine blue staining and TEM examination. A 2 mm section of the regenerated nerve was fixed in 2.5% glutaraldehyde for 2 h, followed by post‐fixation in 1% osmium tetroxide for 1.5 h. The tissue samples were then dehydrated in a graded series of ethanol, embedded in an Epon 812 resin (Ted Pella, Redding, CA, USA), and cut into 1 µm semi‐thin and 50 nm ultrathin sections. While the semi‐thin sections were stained with toluidine blue, the ultrathin sections were analyzed by TEM (JEOL, Tokyo, Japan). Images were acquired from five random fields for each section. The diameter of a myelinated axon and thickness of a myelin sheath were quantified using the Photoshop CS6 software (Adobe Systems). The G ratio refers to the ratio between the axon diameter and fiber diameter. Photographs of five random fields for each ultrathin nerve section were analyzed, and five samples from each group were used for statistical analysis.

### Morphometric Examination of the Reinnervated Muscles

The bilateral triceps surae muscles were harvested from each group at 12 weeks post‐surgery. First, the muscle mass was measured and the wet weight ratio was calculated. Subsequently, it was fixed and embedded in paraffin and cut into transverse sections for Masson's trichrome staining (ab150686, Abcam). Four samples from each group were stained and five randomly selected fields for each specimen were photographed. Thereafter, the CSAs of the muscle fibers were measured using the ImageJ software (Media Cybernetics, Bethesda, Maryland, USA). The percentage of collagen fibers was calculated by dividing the collagen fiber area by the collagen and muscle fiber areas.

### Statistical Analyses

Data are presented as mean ± standard deviation. Statistical differences were determined by performing one‐way factorial analysis of variance and Tukey's post hoc test. GraphPad Prism 6.01 (GraphPad Software Inc.) was used for statistical analysis, and statistical significance was set at *P* < 0.05.

## Conflict of Interest

The authors declare no conflict of interest.

## Author Contributions

Conceptualization: Z.Y., W.H.Y., J.K.X., L.M.B., L.Q. Methodology: Z.Y., W.H.Y., J.M., H.Y., Y.L., X.L. Investigation: Z.Y., W.X.T., D.H.K.C. Visualization: Z.Y., J.X.G., S.X.X. Funding acquisition: L.M.B., L.Q. Project administration: J.K.X., W.X.T., D.H.K.C. Supervision: L.M.B., L.Q. Writing—original draft: Z.Y., W.H.Y., J.K.X. Writing—review & editing: J.K.X., Q.T.Z., P.C.H., L.M.B., L.Q.

## Supporting information

Supporting Information

## Data Availability

The data that support the findings of this study are available from the corresponding author upon reasonable request.
