# Supplementary material for: Magnesium‐Encapsulated Injectable Hydrogel and 3D‐Engineered Polycaprolactone Conduit Facilitate Peripheral Nerve Regeneration
Source: Adv Sci (Weinh). 2022 Jun 2;9(21):2202102. doi: 10.1002/advs.202202102 (PMC9313484; doi:10.1002/advs.202202102)
Supplement: Supplementary file 1 — Supporting Information [file ADVS-9-2202102-s001.pdf]

ADVANCED SCIENCE
Open Access

Supporting Information

for *Adv. Sci.*, DOI 10.1002/advs.202202102

Magnesium-Encapsulated Injectable Hydrogel and 3D-Engineered Polycaprolactone Conduit Facilitate Peripheral Nerve Regeneration

Zhi Yao, Weihao Yuan, Jiankun Xu, Wenxue Tong, Jie Mi, Pak-Cheong Ho, Dick Ho Kiu Chow, Ye Li, Hao Yao, Xu Li, Shunxiang Xu, Jiaxin Guo, Qingtang Zhu, Liming Bian and Ling Qin**

Supporting Information

Supplementary materials and methods;

Supplementary figures;

Supplementary table;

Supplementary Materials and Methods

NMR measurements

The NMR measurements were carried out using a Bruker Bruker AVANCE III 400 NMR Spectrometer at room temperature on samples prepared in D_2O. Calibration was performed using the signals of the solvents, 4.79 ppm for H_2O. The ^{1}H NMR measurements were done by 32 scans and the ^{31}P NMR measurement was done by 512 scans. The modification degrees were calculated by the normalized integration method.

Supplementary figures

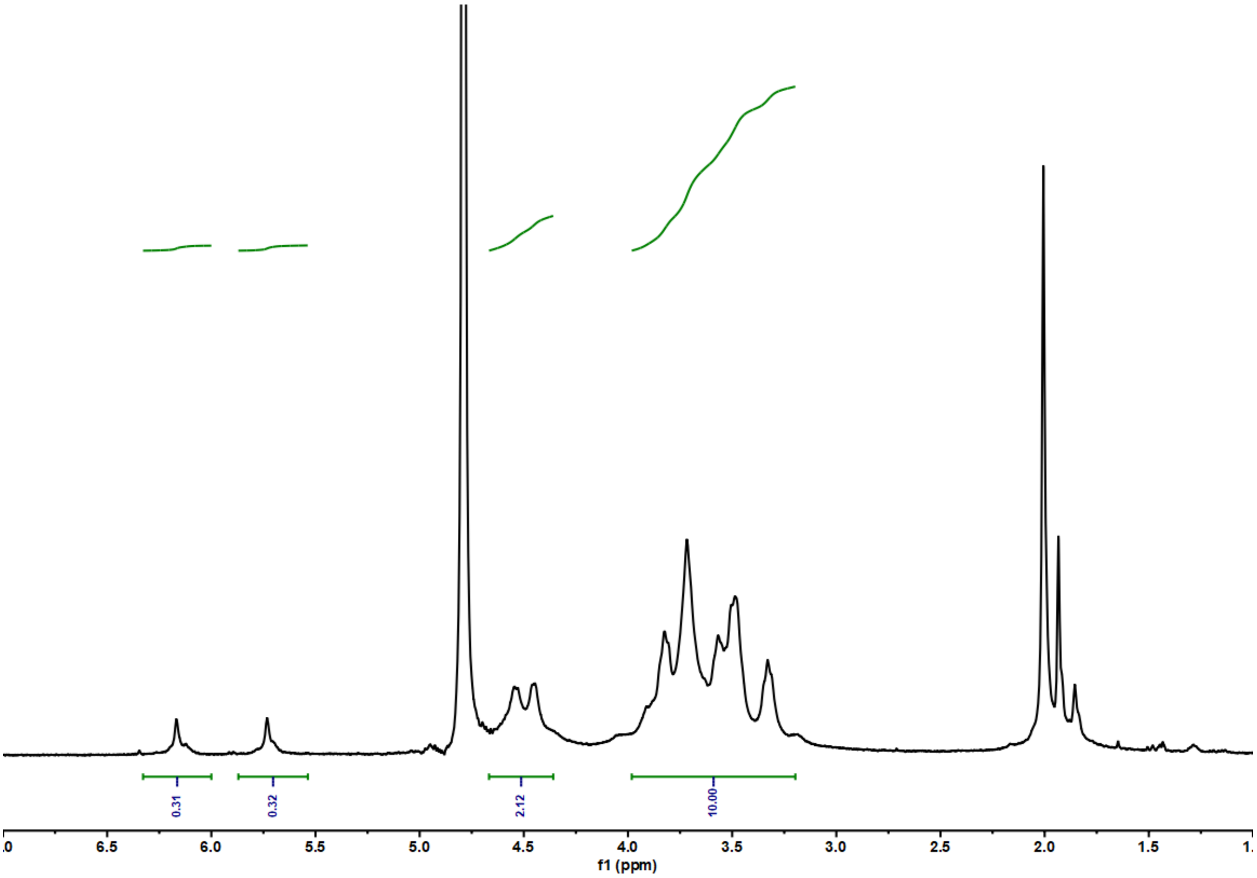

Figure S1 The 1H NMR Spectrum of MeHA. The Substitution degree of methacrylate was calculated as 30%.

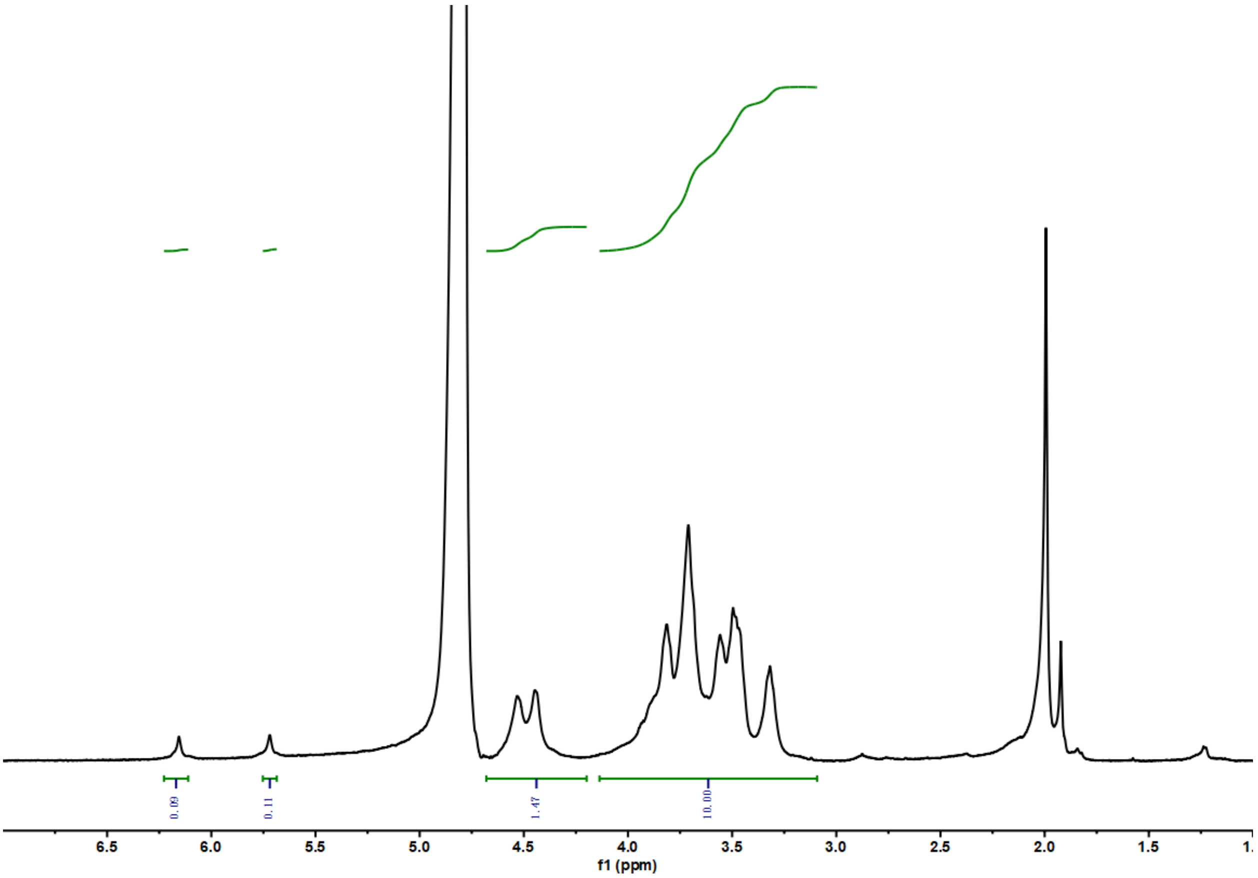

Figure S2 The 1H NMR Spectrum of HA-Pam. The Substitution degree of bisphosphonate was calculated as 20%.

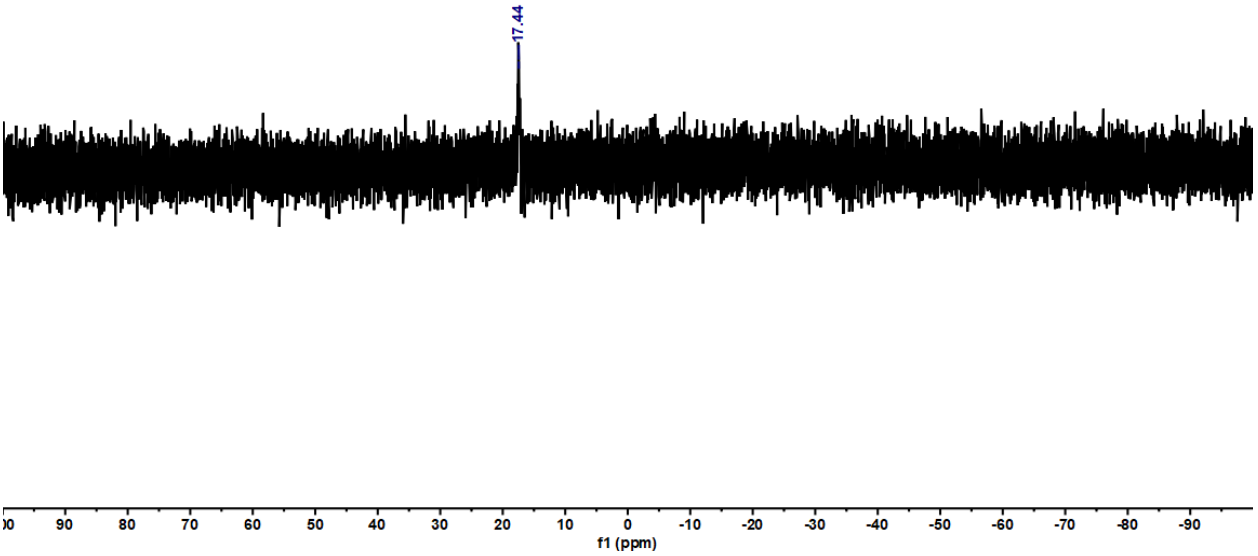

Figure S3 The 31P NMR Spectrum of HA-Pam. The existing peak at 17.44 ppm confirmed the successful conjugation of BP on the HA backbone.

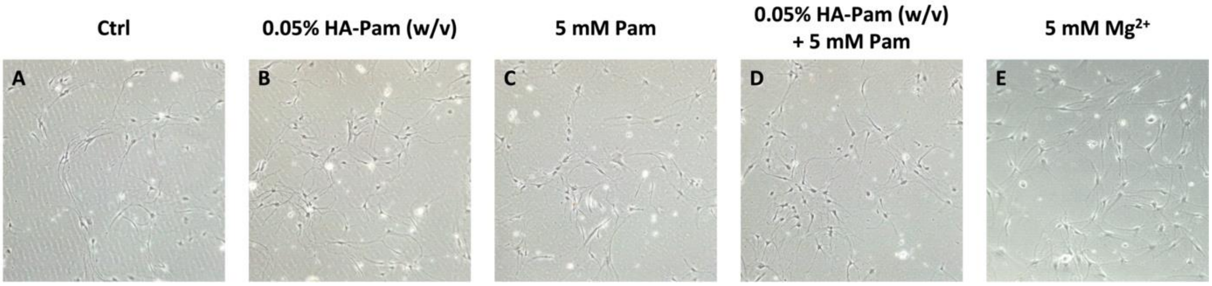

Figure S4 Primary culture of DRG neurons treated with additional Mg^{2+}, HA-Pam and/or Pam.

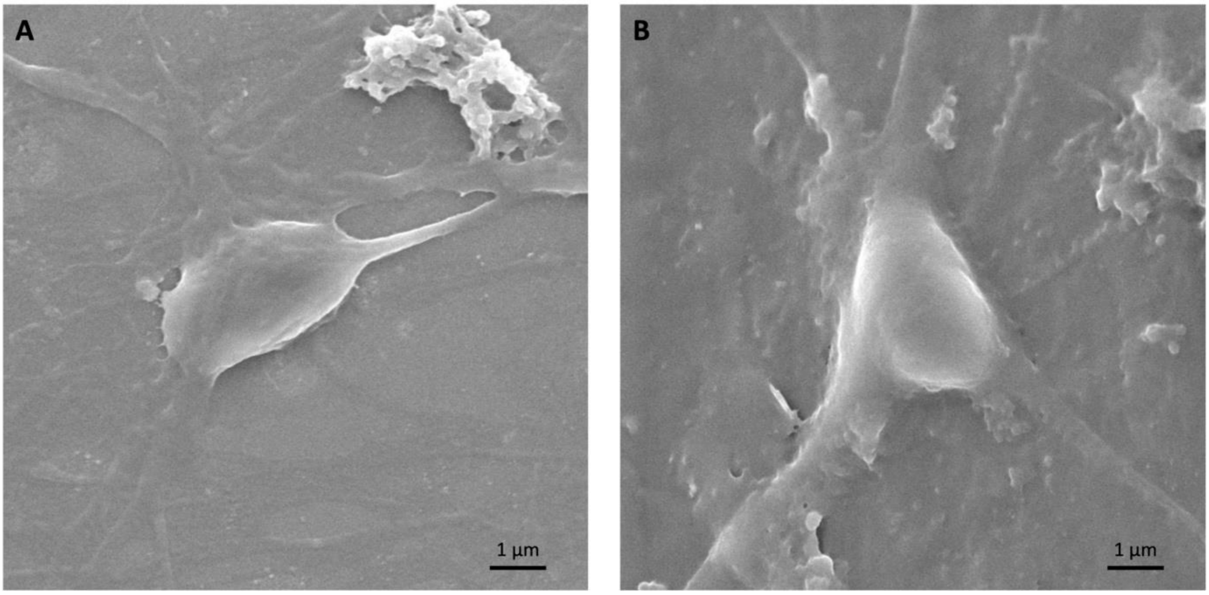

Figure S5 SEM observation of DRG neurons cultured on 3D engineered PCL conduits. A: DRG neurons cultured on general culture dish. B: DRG neurons cultured on 3D engineered PCL conduits.

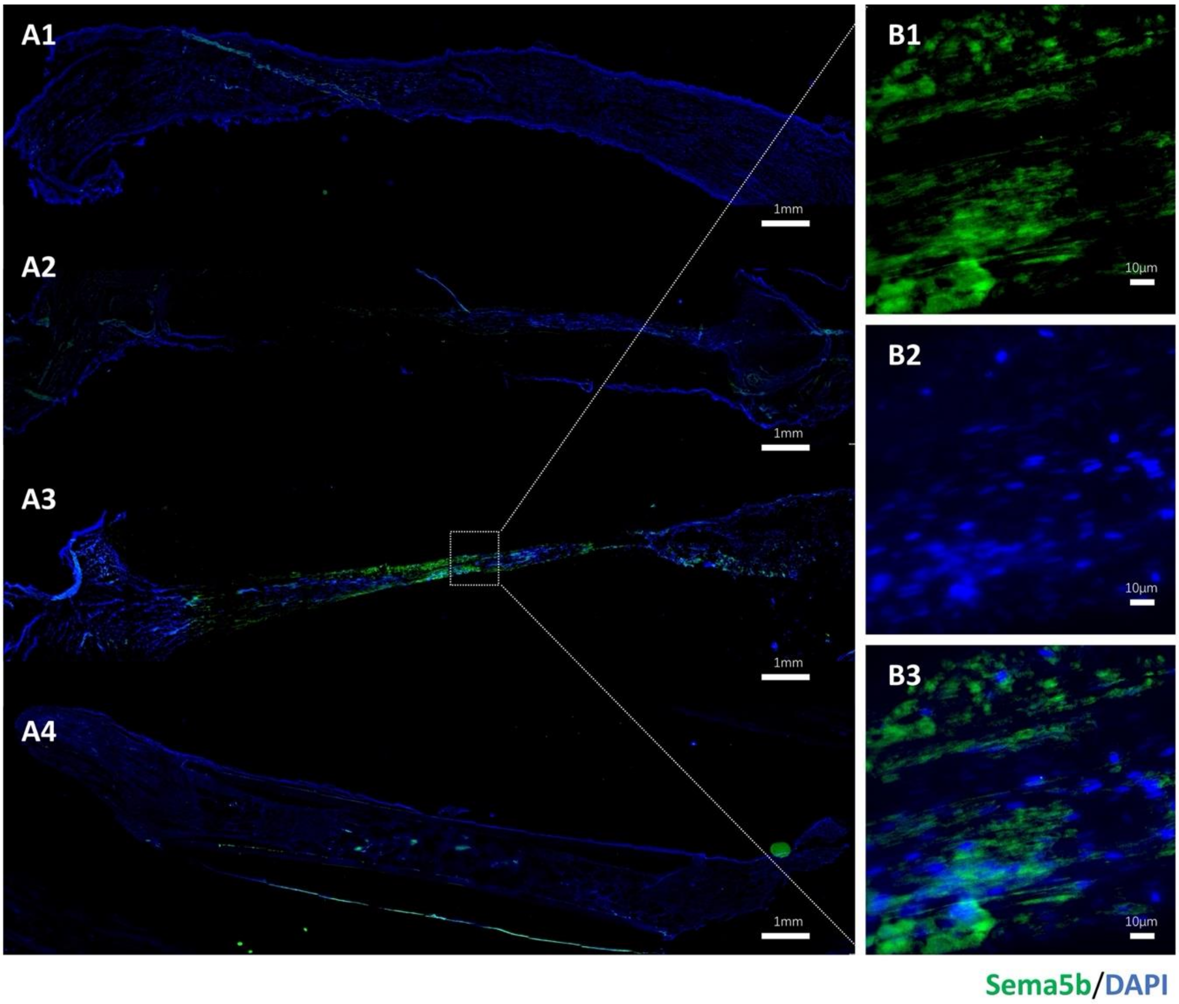

Figure S6 Immunofluorescence analysis of Sema5b expression at early time point.

A: Full-length longitudinal sections of nerve samples demonstrated Sema5b expression. A1-4: Autograft group, PCL nerve conduits group, HA-Pam-Mg hydrogel group, MeHA hydrogel group; B1-3: Sema5b staining of regenerated axons. Timepoint: week 2. Scale bar: 1mm (A1-A4), 10μm (B1-B3).

Supplementary table

Gene	Forward Primer	Reverse Primer
Sema3a	ATATGCAAGAATGACTTTGGAGGAC	AAGGAACACCCTTCTTACATCACTC
Sema3b	GCTGTCTTCTCCACCTCCAG	ACATGCCAGGTCTTGGGTAG
Sema3c	AGACGTGAGACACGGGAATC	TTCATTCAGTTTAACCCTCCTTCC
Sema3d	CTGGATACCCTTTTTGGGTTCAT	AACCAGAATGAGCAGGAAGAC
Sema3e	GCGTCAGTGATGGCTACAGA	CAAAACCCGGACATAATTGG
Sema3f	CTCTTCCAAGAGGCAACAACTG	TTTGCATTGGAATTGAAACCAC
Sema3g	GGGTCCCTAGACCTCCAAGTC	GTCTTTTCCCTTACGGACACA

Sema4a	TGGAGTCTCCTGTGTGTTTTT	CAGCGTGTCAAAGTCcCGG
Sema4b	GGCCCTCTTTGCGCTTAACAG	TGTAGTTTTGACAGTCACGCTT
Sema4c	GAGATGTGGTGGAACCTTGTG	CAGGGTCAGCGTCAGGAAGTC
Sema4d	TCAAAGCAGACGGACTGCCTA	CCCCCACCATGACCGATGTGTA
Sema4f	TTATGCTTGCGAGTGTCAGG	GAGGAGAGTGAGGGATGCTG
Sema5a	GGACCCCTGTCAACATCTCTG	CCTCTGTCTTCCTACTTCCAGC
Sema5b	GAAGCCGTGGGTCTGTAACTT	GGAGGGATACATTGGCTAG
Sema6a	ACAGCCTGCCCCCGAAAGT	AGCTCCTCTTATATTCTAGCCC
Sema6b	GCTTATGCAGAATGGCTGGAC	GCGTGTGGGTGTGTTGTG
Sema6c	TCAGCCTCCCGATCCATCC	ACAAGCACAGGACACCAAGAG
Sema6d	AATATCCGGTTTTTAGAGGACGC	TGCTTGCCTTTCATAGCACAAT
Sema7a	GCTCCATTGCAGAAGGTTTC	GTTGAGCCTCACGGAGGTAG
Slit1	GCCTGGAGCTGAACGGTATC	GGC ATC GGG TGC AAT CTC
Slit2	TCAACGCCTTCTCCTACA	GCACTTCACCACCTTCTC
Slit3	TGCCCCACCAAGTGTACCT	CGCCTCTCTCGATGAGGCT
Netrin1	GCTTCCAAAGGAAAACTGAA	CTTCCACCAGTCCCCTGCTT
Netrin3	GCCGACCCCTGCTATGATG	GTTGCAGCAGATCGGAGCG
Netrin4	CCCATGTACTGGCGGAGA	GCGGAGGTTGGTGATCTTC
NetrinG1	TCAAGATTCCTGTCGATACATGC	ATGTCCCCACACAAAACGGTA
NetrinG2	GTGATGCGCCTGAAGGATTAT	CTGCACAGGTATGGGTTCTC
Efna1	CTTCACGCCTTTGAGCTTGGGC	TGGGGCGTCTGAGTGATTTTGCC
Efna2	CGATACGCGGTCTACTGGAAC	CCAGGTAGTCTTTGATGCTCA
Efna3	CGTGCATGTGAACGTGAAC	GTACCGCTGGAACTTCTCGGA
Efna4	CAGGTTGCTACGAGGAGATG	GGGCTGTCATAATGTGGGCAG
Efna5	TCCAGAGGGGTGACTACCAC	GGCACTGTACCCATCAAAATTCA
Efnb1	TGTGGCTATGGTCGTGCTG	TCTTCGGGTAGATCACCAAGC
Efnb2	TTGCCCCAAAGTGGACTCTAA	GCAGAGGGGTATTATCCTTC
Efnb3	GAGGGAGGTTACGTGCTTTAG	GGGCTGTATTCCTGGAACTTGAT
BDNF	CGTGGGGAGCTGAGCGTGTG	GCCCCTGCAGCCTTCCTTC
VEGF	GCCGCAGGAGGCAAACCGAT	TGGCGGGCTCCTCTCCCTT
NGF	TGCATAGCGTAATGTCCATGTTG	CTGTGTCAAGGGAATGCTGAA
GAPDH	ATGACTCTACCCACGGCAAG	CATACTCAGCACCAGCATCAC

Table S1 Primer sets designed for quantitative real-time PCR.
